# Panera: An innovative framework for surmounting uncertainty in microbial community modeling using pan-genera metabolic models

**DOI:** 10.1016/j.isci.2024.110358

**Published:** 2024-06-22

**Authors:** Indumathi Palanikumar, Himanshu Sinha, Karthik Raman

**Affiliations:** 1 Department of Biotechnology, Bhupat Jyoti Mehta School of Biosciences, Indian Institute of Technology (IIT) Madras, Chennai 600 036, India; 2 Centre for Integrative Biology and Systems mEdicine (IBSE), IIT Madras, Chennai 600 036, India; 3 Robert Bosch Centre for Data Science and Artificial Intelligence (RBCDSAI), IIT Madras, Chennai 600 036, India; 4 Department of Data Science and AI, Wadhwani School of Data Science and AI, IIT Madras, Chennai 600 036, India

**Keywords:** Microbiology, Bioinformatics, Computational bioinformatics, Systems biology

## Abstract

Utilization of 16S rRNA data in constraint-based modeling to characterize microbial communities confronts a major hurdle of lack of species-level resolution, impeding the construction of community models. We introduce “*Panera*,” an innovative framework designed to model communities under this uncertainty and yet perform metabolic inferences using pan-genus metabolic models (PGMMs). We demonstrated PGMMs’ utility for comprehending the metabolic capabilities of a genus and in characterizing community models using amplicon data. The unique, adaptable nature of PGMMs unlocks their potential in building hybrid communities, combining genome-scale metabolic models (GSMMs) and PGMMs. Notably, these models provide predictions comparable to the standard GSMM-based community models, while achieving a nearly 46% reduction in error compared to the genus model-based communities. In essence, “*Panera*” presents a potent and effective approach to aid in metabolic modeling by enabling robust predictions of community metabolic potential when dealing with amplicon data, and offers insights into genus-level metabolic landscapes.

## Highlights

• “*Panera*” tackles uncertainty in *in-silico* community modeling using amplicon data
• Pan-genera metabolic model explores genus metabolism, revealing functional landscapes
• Hybrid models make metabolic predictions comparable to GSMMs in *in-silico* communities

## Graphical abstract

**Figure:**
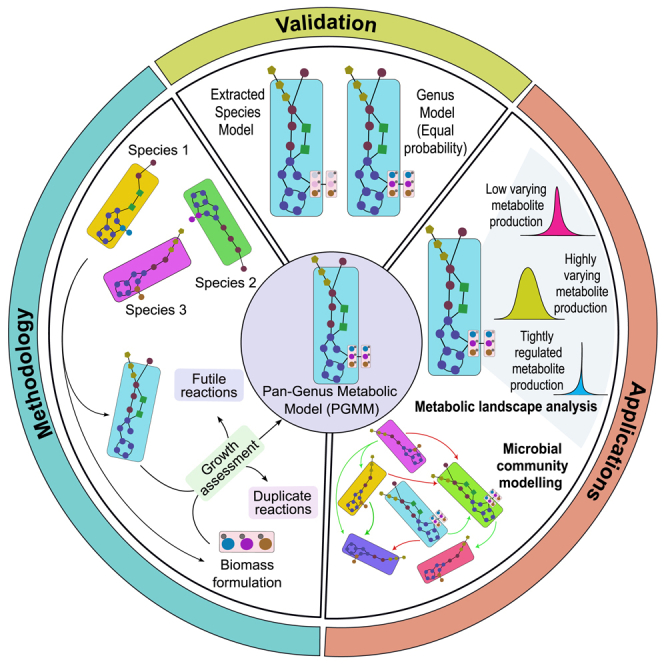

## Introduction

The past decade has witnessed unprecedented growth in metagenomics research, highlighting the significance of microbial communities in diverse ecosystems. These microbial communities thrive in environments ranging from host-associated systems^1^^,^^2^^,^^3^ and environmental conditions^4^ to even extreme environments like hot springs^5^ and the ocean floor.^6^ Unveiling the composition of these microbiomes relies on techniques like amplicon sequencing, which targets specific gene segments (often the 16S rRNA gene), and shotgun metagenomics, which sequences entire genomes present in a sample.^7^ By analyzing the compositional and functional profiles, the microbial habitats and their activities within an ecosystem can be inferred.^8^ However, a deeper understanding of the intricate interactions within the microbial community as well as with its host and environment is still lacking.

Genome-scale metabolic models (GSMMs) have emerged as a powerful tool for comprehending the complex relationship between an organism’s genetic makeup (genotype) and its observable traits (phenotype).^9^^,^^10^ These models are instrumental in simulating the physiological behavior of the biological organisms *in silico* and analyzing their responses to varying nutrient conditions and genetic modifications.^11^^,^^12^ Prior research analyses have harnessed GSMMs to reconstruct microbial communities *in silico*, providing insights into their metabolic dependencies and functionalities.^13^^,^^14^^,^^15^^,^^16^ GSMMs have proved to be a valuable and standard resource for *in-silico* reconstruction of microbial communities^17^^,^^18^ and are publicly available in databases such as AGORA, AGORA2, and BiGG.^19^ The metabolic characterization of these microbial communities opened avenues for diverse applications, including biomarker identification,^20^ disease classification,^21^^,^^22^ host-microbiome interaction exploration,^23^ and personalized treatment regimen development.^24^ However, a significant limitation of these simulations lies in their reliance on GSMMs derived from whole genomes. These approaches are widely used but presuppose complete knowledge of all individual species within a community, which is often not achievable from amplicon sequencing. The recent surge in microbiome research, particularly studies employing shotgun metagenomics, has opened avenues for enriching GSMMs using metagenome-assembled genomes (MAGs). GSMMs reconstructed from MAGs hold promise for building microbial community models only when shotgun metagenomics data are available for microbiome characterization.^15^^,^^25^^,^^26^ Given the limitations of amplicon sequencing in generating MAGs, existing model reconstructions deposited in public databases such as AGORA, BiGG, KBase, and CarveMe become crucial resources for building and analyzing microbial communities characterized through amplicon sequencing data.

A major limitation of the GSMM-based modeling approach stems from the inherent ambiguity of using 16S rRNA sequencing data. Typically, only a short segment (250–500 bps) of the 16S rRNA gene is analyzed, representing just a fraction of the entire gene (1,500 bp). This limited information often restricts taxonomy assignment to the genus level, for more than half of the sequencing read.^27^ Given the widespread availability of 16S datasets, there is a pressing need for novel frameworks that can leverage this genus-level information along with species-level data. Pan-genus models, a genus-level metabolic model that captures the collective metabolism of all the species within a genus and a GSMM equivalent for genus, emerge as a promising solution to model microbial communities characterized by incomplete taxonomic information.

It is essential to appreciate the role of pan-genus models in elucidating the unique metabolic capabilities and physiological characteristics of a genus. To date, two major approaches exist for pan-genus model reconstruction. The first involves building a pan-genome, which combines the genome of all species within a genus.^13^ This pan-genome then serves as the basis for draft model reconstruction using tools like KBase^28^ and CarveMe.^29^ For instance, the pan-genome models of *Propionibacterium*,^30^ *Escherichia*,^31^ and yeast^32^^,^^33^ have been reconstructed from their respective pan-genomes to investigate diverse and shared metabolic traits within a genus and understand strain-specific adaptations. However, this approach often necessitates extensive manual curation to address gaps and ensure model consistency.

An alternative, more streamlined approach relies on existing curated species-specific GSMMs from databases like AGORA^34^^,^^35^ and BiGG. This approach circumvents the challenges of manual curation,^30^ making it more scalable for reconstructing models for multiple genera. Prior studies have demonstrated the utility of the panModels built using the “createPanModels” routine from the Microbiome Modeling Toolbox (MMT),^36^ in studying the alteration in the metabolism of the human microbiome under different disease conditions.^21^^,^^37^^,^^38^^,^^39^

**Figure 1:**
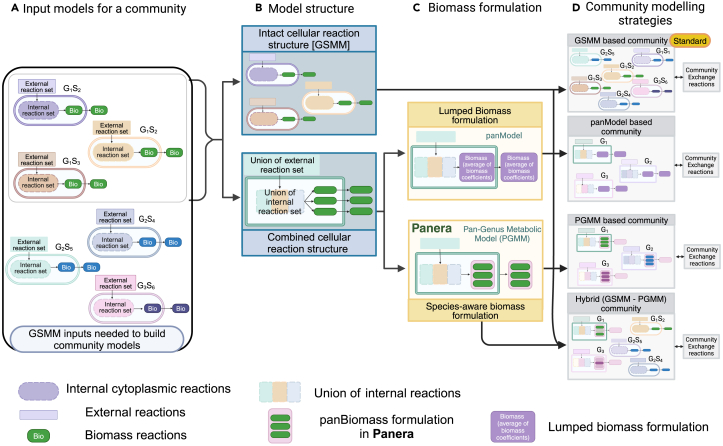
Overview of model reconstruction for different microbial community modeling approaches The figure depicts the key steps involved in reconstructing different models (GSMM, PanModel, and PGMM) and their application in modeling microbial communities (Created with BioRender). (A) illustrates the GSMMs as input for constructing community models using species-level information; (B) depicts the generation of genus models by aggregating species models and retaining only the unique reactions within the combined model; (C) shows how the combined reaction structure is further defined by a biomass equation to create a functional model and illustrates how PanModels differ from pan-genus metabolic model (PGMM). PanModels utilize a lumped biomass approach, averaging the biomass reactant and product coefficients. In contrast, the PGMMs formulated by *Panera* incorporate species information into the model by defining the biomass as a combination of species abundance coefficients and individual species biomass reactions; and (D) illustrates the reconstruction of different community models. GSMM-based communities serve as the benchmark, while hybrid communities are proposed for modeling microbial samples sequenced using 16S rRNA sequencing, which typically provides a mixture of species and genus-level information (see also Figure S4; Tables S1 and S2).

However, existing tools like “createPanModels” suffer from significant limitations. These limitations hinder the ability of the models to fully exploit the potential of 16S rRNA sequencing data. This tool relies on a simplistic merging of existing models, creating a lumped biomass (as demonstrated in Figure 1) and cannot leverage species-specific information in 16S data. Furthermore, these tools frequently restrict taxonomic input at either the genus or species level. Given the inherent nature of amplicon sequencing data, which often provides a mix of species and genus-level taxonomic information, disregarding either level of resolution can lead to inaccurate metabolic predictions.

In this study, we propose a unique framework to address two critical challenges in microbial community characterization: (1) uncertainty associated with taxonomic assignment in 16S rRNA sequencing data and (2) scarcity of representative genus-level models. Our framework strives to unlock the full potential of pan-genus metabolic models (PGMMs) for improved characterization of microbial communities. Our approach involves the generation of a comprehensive repository of flexible PGMMs constructed by harnessing curated GSMMs. We assess the applicability of these reconstructed PGMMs in two key aspects: (1) modeling microbial communities characterized by uncertain taxonomic information and varying resolution and (2) investigating the intricate metabolism within individual genus members to understand their unique functional characteristics. By overcoming the limitations of current PGMM construction methods and the inherent resolution constraints of 16S rRNA sequencing, our method paves the way for significantly more accurate predictions of the metabolic capabilities of microbial communities.

## Results

In this work, we present the “*Panera*” algorithm, designed to tackle uncertainties in metabolic function prediction arising from the limitations of 16S rRNA sequencing data. While 16S sequencing-derived taxonomic data offers valuable insights, it often lacks species-level resolution, a key input for accurate community metabolic modeling. Traditionally, only species-level data are utilized in microbial community modeling, neglecting approximately 40% of the information available at the genus level. In addition, an alternate approach, building community models using genus-specific models with aggregated genus-level taxonomic data, not only discards valuable species information but also reduces the accuracy of community-level metabolic predictions. *Panera* addresses this challenge by generating species-aware genus-level metabolic models and integrating them into species-level GSMMs to construct an *in-silico* representation of a microbial community. We achieve this by reconstructing PGMMs from existing GSMMs. These PGMMs capture unique reactions specific to each genus and incorporate the species composition through flexible biomass formulation. Subsequently, we evaluated the ability of PGMM to represent both individual species and entire genera. Furthermore, the reconstructed PGMMs are shown to be a valuable tool for exploring the metabolic potential of individual genera, identifying context-dependent functional similarities between genera, and modeling diverse microbial community scenarios. Our analysis reveals that these hybrid models, incorporating both species and genus information, demonstrate a significant reduction (46%) in predicting metabolic capabilities compared to the lumped model-based communities. This improvement underscores the efficacy of *Panera*-derived hybrid models in enhancing the accuracy of microbial community modeling and their functionality prediction.

### PGMM can be a representative of both genus and species

**Figure 2:**
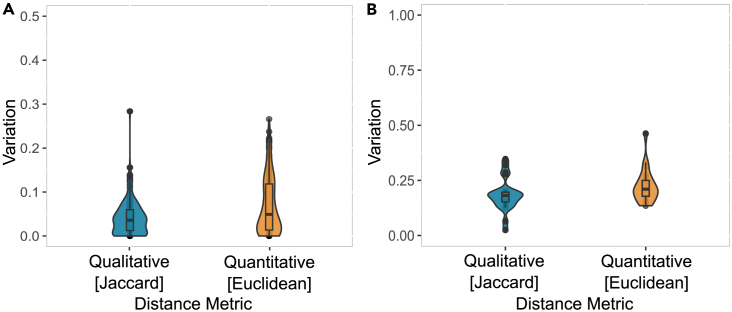
PGMM can be a representative of both genus and species Qualitative (variation in the active exchange metabolites in the model using Jaccard distance) and quantitative (differences in the maximum production flux of the exchange metabolites within the model) variation in the metabolic potential prediction of (A) individual species extracted from PGMM-*Panera* models in comparison to GSMM (the variation is reported for 150 individual species) and (B) equal-species abundance implied PGMM in comparison to GSMM-based genus models (the variation is reported for 20 different genera) (see also Figure S5; Tables S3 and S4).

To assess the ability of PGMMs to retain the functionalities of individual species models, we examined them against the respective species GSMMs from the AGORA database (detailed information on the species models used and the variations observed are provided in Table S3). Species representation by the *Panera* PGMM was evaluated by comparing the net maximum production/consumption potential of exchange metabolites with the flux predicted by the GSMM using flux variability analysis (FVA) (Figure 2A). A significant flux difference was defined as a ratio exceeding 10% between the maximum exchange metabolite flux in the GSMM and the species model derived from the PGMM.^40^ This analysis showed nearly 8.7% of exchange reactions have significantly different flux values. Qualitative analysis, focusing on the number of metabolites produced within a model using Jaccard distance, revealed minimal variation between the models, with an average of 4%. Conversely, quantitative analysis investigating variations in metabolite production/consumption rates using Euclidean distance showed a moderate variation ranging between 5 and 10% in the fluxes of exchange metabolites.

We further investigated the capability of PGMMs to represent the metabolic potential of a genus. This was evaluated by comparing PGMMs to genus-level models built using GSMMs (Table S4). To simulate an equal presence of all species within a genus, we modified the PGMM using a species probability vector where all species have a uniform proportion summing to 1. Subsequently, the genus-level model was reconstructed by integrating the corresponding GSMMs at equal abundances through a compartmentalization approach. In contrast to individual species models, substantial variability in both qualitative and quantitative analyses was observed between the genus representation by PGMM and the GSMM-based genus model (Figure 2B). Analysis on maximum exchange metabolite production flux revealed an average difference of 38% between the models. Additionally, an 18% variation was observed in predicting the number of metabolites produced.

### Metabolic landscape analysis reveals a fascinating context-specific metabolic similarity between genera

The inherent flexibility of *Panera*-derived PGMMs allows for the investigation of metabolic production landscape within a genus, providing insights into its overall metabolic capabilities and potential metabolic niche development within a genus. For instance, switching from tightened control of reaction flux in the genus model containing (*n*-1) species to broader flux bandwidth in the genus model containing *n* species suggests that the addition of nth species contributes to the metabolic pathway diversification that contributes to the production of that particular metabolite. We categorized the observed bandwidth into four distinct groups: high variability, low variability, tight regulation, and no production. Tight regulation of specific metabolites across all species within a genus, regardless of species composition, suggests conserved flux maintenance and potential essentiality for survival under the simulated condition. Conversely, metabolic production with a broader flux range indicates the varying impact of individual species on the overall production potential, i.e., the metabolite production may be dependent on the cross-feeding interactions or presence of other species.

Interestingly, mapping the metabolic flux bandwidth of exchange metabolites across all genera revealed greater metabolic similarities among opportunistic pathogens compared to commensal genera (Figures S2A and S2B: heatmap of the metabolic bandwidth for exchange metabolites across genera).^41^^,^^42^^,^^43^^,^^44^^,^^45^ This observation aligns with the distance trees constructed based on the metabolite similarity (Figure S1: distance tree based on metabolites similarity of a genus) and reaction similarity (Figure S1: distance tree based on reaction similarity of a genus), which rely on the presence or absence of these entities. These trees demonstrate similar metabolic potential within clusters of organisms including opportunistic pathogenic clusters, such as cluster-I (*Staphylococcus*, *Streptococcus*, *Shigella*, and *Serratia*), cluster-II (*Haemophilus*, *Helicobacter*, *Klebsiella*, and *Gemella*), and commensal genera (*Blautia*, *Bifidobacterium*, *Bacillus*, and *Bacteroides*). Furthermore, flux bandwidth analysis offers insights into genus-specific characteristics. For instance, the distinct clustering of *Bacteroides* and *Prevotella* observed on the reaction and metabolite similarity-based trees is challenged by the flux bandwidth-based tree (Figure S1). This suggests a potential shared metabolic regulation between these genera, even with some dissimilarity in their reaction and metabolite profiles. A similar pattern is observed for *Shigella*, *Enterobacter*, *Klebsiella*, and *Haemophilus.* Notably, over 70% of the clustering patterns based on flux bandwidth types remained consistent across different dietary conditions (i.e., on both European and Mediterranean diets).

**Figure 3:**
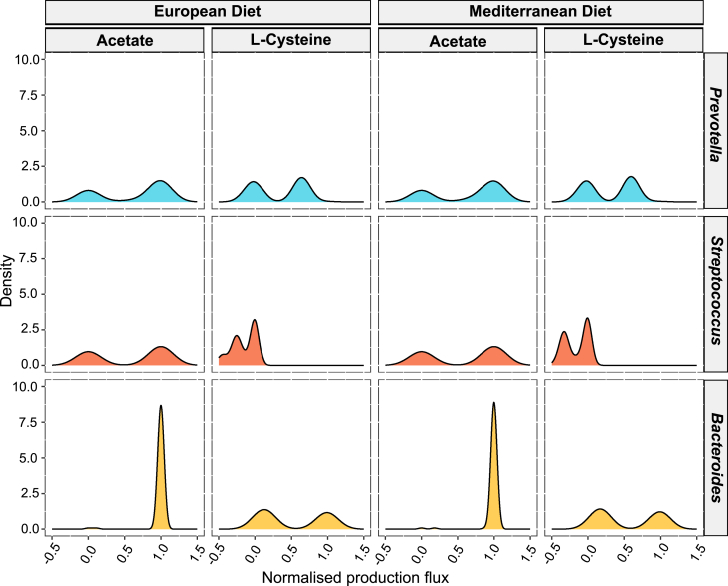
Metabolic flux bandwidth analysis of acetate and L-cysteine production by different genera Metabolic flux bandwidth of acetate and L-cysteine production across various genera is estimated with 120 different species combinations under two dietary conditions: European (EU) diet (left panel) and Mediterranean diet (right panel). The analysis reveals tighter regulation of acetate production in *Bacteroides* under both diets, while *Streptococcus* and *Prevotella* exhibit greater production variability across the diets (See also Figures S1, S2, and S6A).

Our analysis revealed that the metabolic flux bandwidth of a specific metabolite can vary among different genera, and this variation appears to be indirectly related to metabolite regulation. For example, the flux range of L-cysteine observed in *Bacteroides* and *Prevotella* was notably wider than that in *Streptococcus* (Figure 3). However, a bimodal distribution of the flux range, regardless of its magnitude (high or low), suggests distinct control mechanisms employed by the species in these genera for cysteine production. Additionally, the fluctuating flux range distribution for acetate and L-cysteine across these three genera highlights the influence of species composition on metabolic control. Further investigation of the metabolic flux bandwidth for each exchange metabolite within these genera (Figure S2) revealed stricter regulation across different genera for metabolites associated with inorganic ion metabolism, such as zinc, copper, and magnesium. This may be attributed to the limited micronutrient requirements for these organisms.

We observed that the cofactor group, which includes quinone, glutathione, folate, and riboflavin, showed a robust regulation in most of the genera, except for the genus cluster containing *Shigella*, *Escherichia*, *Enterococcus*, *Klebsiella*, and *Haemophilus* under both dietary conditions. On the other hand, this specific genus cluster displayed robust regulation for other cofactors like reduced glutathione. These observations suggest that different genera might have unique cofactor requirements essential for their specific functionalities. In contrast, amino acid production fluxes, another class of metabolites, exhibited greater variability than the other metabolites group. Notably, commensals displayed weaker regulation over amino acid production compared to other genera. Most of the observed results remained consistent under both simulated dietary conditions. Consequently, we detected higher similarity in metabolite-level clustering between European and Mediterranean diet conditions. Overall, this study leverages the flexibility of PGMMs to explore the metabolic potential and regulatory landscapes within microbial genera. The observed variations in metabolic flux bandwidth provide valuable insights into the functional characteristics and potential niche adaptations of different genera.

### PGMM captures better metabolic information than the lumped genus model in community modeling

We investigated the applicability of PGMMs in characterizing microbial communities through metabolic modeling using both the metagenomic data generated synthetically and data from colorectal cancer (CRC) studies. We employed synthetic microbial abundance data for various community sizes (*n* = 10 and 50). Four distinct *in-silico* community types were reconstructed, and their predicted metabolic outputs were compared to those obtained from widely used GSMM-based communities (as demonstrated in Figure 1).

**Figure 4:**
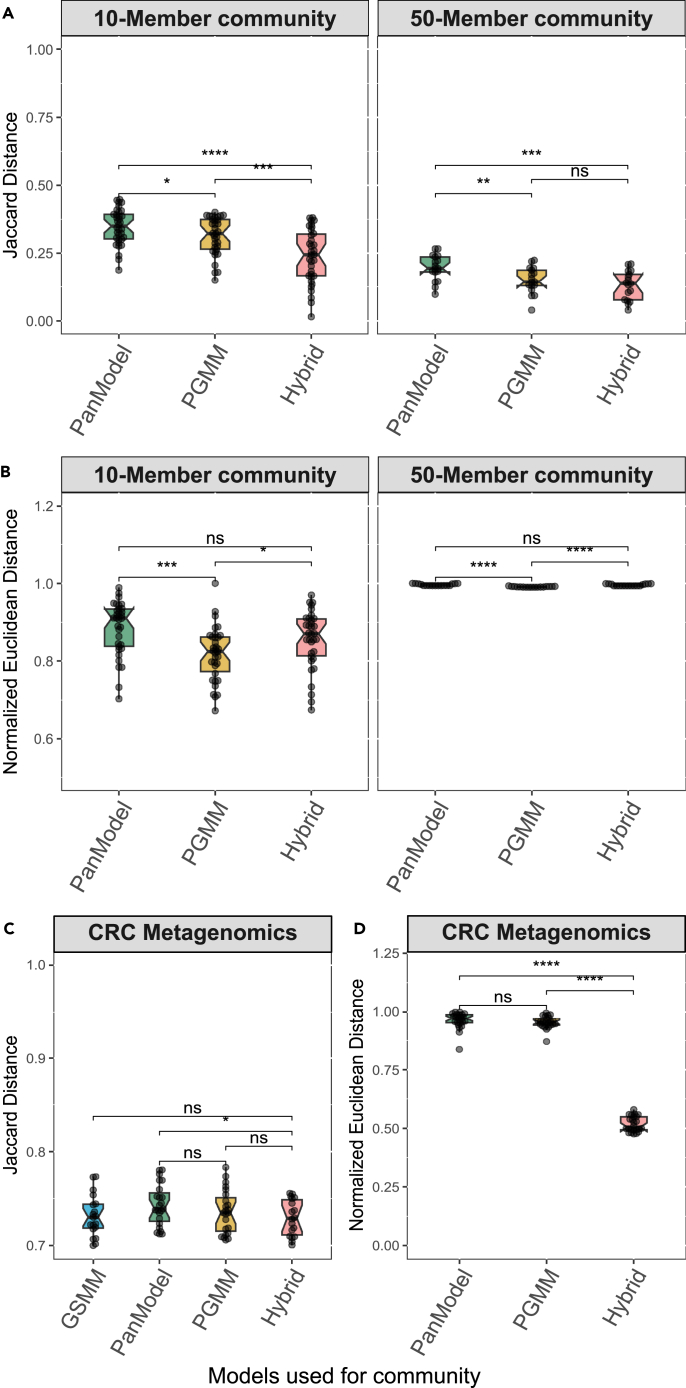
Hybrid models in community modelling improve the prediction of metabolic potential The figure presents a comparative analysis of qualitative (Jaccard distance) and quantitative (normalized Euclidean distance) differences in the metabolic potential between various community models and GSMM-based communities for two datasets: synthetic dataset (A and B) and CRC metagenomics dataset (C and D). The analysis is employed for different community types, including PGMM (*Panera*-derived pan-genus metabolic model), PanModel (pan-genus metabolic model constructed with “CreatePanModels” in the CobraToolBox suite), and hybrid communities (combining GSMM and PGMM). The variation is evaluated in relation to GSMM-based community models. The reported significance is calculated based on the paired t-test and the level of significance is represented as follows: ns, not significant; ∗*p* ≤ 0.05; ∗∗*p* ≤ 0.01; ∗∗∗*p* ≤ 0.001 and ∗∗∗∗*p* ≤ 0.0001 (See also Figures S3 and S6B; Tables S5, S6, and S7). (A) Highlights qualitative differences in metabolic diversity and (B) quantitative differences (variation in metabolite production flux) when compared to GSMM-based communities for both 10 (no. of models, *n* = 34) and 50-member synthetic communities (no. of models, *n* = 15). (C) Demonstrates the variance in metabolic production diversity within a community when compared to metabolomics study in the CRC metagenomics dataset (no. of models, *n* = 24). (D) Displays the differences in metabolic production flux within a community concerning GSMM-based communities in the CRC metagenomics dataset.

Qualitative variation analysis revealed that *Panera*-derived PGMM models, when implemented within communities, captured a broader range of metabolic information (metabolic information is defined as the functional capability of the microbial community that can be analyzed using constraint-based modeling approaches), i.e., the predicted metabolite production capacity has lesser error while comparing with PanModels for a smaller community (Tables S5B and S6B). Hybrid models showed improved metabolic output predictions from the communities. The difference between PGMM and PanModel became statistically significant with increasing community size (Figure 4A). The uptake metabolic potential is captured significantly better in hybrid communities than PanModel and PGMM-based communities (Tables S5B and S6B; Figure S3). Furthermore, quantitative variation analysis demonstrated that PGMM-based communities offered more accurate predictions of metabolic capabilities compared to PanModel-based communities when benchmarked against GSMM-based microbial communities (Figure 4B; Tables S5C and S6C). Notably, no significant variations were observed in uptake fluxes, representing the community’s consumption capabilities (Figure S3). This analysis suggests that even minor variations in nutrient uptake within the model can lead to substantial changes in predicted production fluxes. Interestingly, while the uptake metabolic flux may be similar across all community models, the metabolic production profiles for the same community differ based on the community type. This observation potentially points toward the inherent differences in the model structure of each community model. In conclusion, our findings indicate that PGMM-based communities and hybrid communities can effectively capture the qualitative metabolic potential of GSMM-based communities than PanModel-based communities. This showcases the potential of PGMM as a valuable alternative to existing PanModel for community metabolic modeling from genus-level data.

### Hybrid models in community modeling improve the prediction of metabolic potential

We subjected microbial communities, generated using CRC-metagenomics data, to a comprehensive analysis encompassing both qualitative and quantitative variations across different modeling approaches. Comparisons were drawn against available metabolomics data for validation. Given the higher computational demand associated with analyzing the metabolic potential of the microbial communities through different modeling strategies, we restricted the analysis to two different sets of around 20 samples each. Both the sets yielded consistent results, which are presented in detail in Tables S7A–S7H. We assessed the qualitative variation by comparing the net metabolic output detected in the metabolomics data with the net metabolic potential produced by the various community types (Figure 4C; Table S7B). Intriguingly, a comparable set of secreted metabolites were captured by the communities built from PGMM, PanModels, and hybrid models (GSMM along with PGMM) when compared to the standard GSMM-based microbial communities. The comparison of the error metrics and their statistical significance revealed that the predictions from hybrid models exhibited a closer alignment with those from GSMM-based communities (Tables S7B and S7E), Conversely, predictions from PGMM-based and PanModel-based communities deviated significantly from the standard GSMM communities. This observation indicates that hybrid models offer accurate metabolic output predictions compared to PanModel-based communities.

To quantitatively evaluate the alteration in the production flux of exchange metabolites, we reported the comparison with GSMM-based communities since the metabolomics data were presented in terms of metabolite concentration along with the significance of the prediction error (Table S7C). No significant differences were observed between PGMM and PanModel communities, with variations falling within a narrow range of 0.2–0.5% (Figure 4D). However, hybrid community models (using both GSMM and PGMM to build a community) demonstrated a 46%–57% reduction in error rate for functional predictions when compared with the maximum flux values of the exchange metabolites from a community (Tables S7C and S7F). Overall, the evaluation of metabolomic analysis outcomes against the predicted metabolic abilities of the various community models insinuates that hybrid models exhibit comparable prediction accuracy as GSMM with minimal qualitative and quantitative disparities.

## Discussion

Metagenomic sequencing technologies, particularly the cost-effective 16S rRNA sequencing, have significantly advanced our comprehension of microbial ecosystems, specifically regarding their compositional and functional dynamics. Nevertheless, the fundamental limitation of acquiring taxonomic assignments at finer taxonomic levels in amplicon sequencing poses a challenge in building communities and predicting community functionality and metabolic interactions using constraint-based *in-silico* microbial community modeling. To address the issue, the PGMM is employed as a valuable alternative to GSMMs for constructing microbial communities and studying their functionalities and dynamics. However, reconstructing high-quality PGMMs from pan-genomes is time-consuming and requires manual curation. This limitation prompts the researchers to explore an alternative approach using existing curated GSMMs. Yet, models generated from one such existing tool, MMT, struggles with accommodating both the genus and species compositionality in a single data and representing species within a genus, which restricts their utilization in community modeling that necessitates the incorporation of both genus and species information from amplicon sequencing. Consequently, the entire potential of metabolic modeling in microbial community analysis remains underutilised.

To address these challenges, we introduce our method, “*Panera*,” which presents a unique and adaptable framework for generating PGMMs (Figure 1). The primary aim of *Panera* is to reduce uncertainties in assessing the metabolic capabilities of a microbial community while using uncertain taxonomic information from 16S amplicon sequencing data. This framework constructs a comprehensive model by integrating all unique reactions from individual species-specific GSMMs within a genus and their respective metabolic data. The “panBiomass” equation, which represents a linear combination of species biomass equations, is then incorporated into the model to obtain a PGMM. The components in PGMM are “species-aware” as opposed to the PanModels built from MMT, which generates a new biomass reaction by averaging the coefficients of the reactants and products involved in the biomass equation. The updated biomass formulation (an objective function) subsequently presents the flux distribution of the PGMM. In addition, the *Panera* algorithm further enhances PGMMs by introducing flexibility, i.e., allowing users to tailor the model to the specific input parameters (microbial species abundance data). We rigorously tested *Panera* PGMMs while predicting the metabolic capabilities of individual species and the collective abilities of genera. These models were evaluated for their primary application in simulating *in-silico* microbial communities, particularly in comparison to conventional GSMMs and investigating the metabolic potential and inter-species/genus communication in a community. Additionally, PGMM showcased their unique utility in exploring the metabolic landscape of genera.

Our analyses demonstrated that PGMMs effectively capture the metabolic activities of individual species GSMMs, as indicated by comparable qualitative and quantitative metabolic flux predictions. However, disparities arose when comparing PGMMs to GSMM-based simulations while analyzing the equal species probability genus-level models. These discrepancies could be attributed to the structural differences between the models. PGMMs exclusively comprise unique reactions of a genus in a single compartment, while GSMM-based genus-level model adopt a compartmentalization approach^46^^,^^47^ that combines all species models via an extracellular compartment while keeping the internal species reactions intact. Despite both models utilizing a similar objective formulation, which involves a linear combination of species proportion and species biomass, variations emerged when simulating a community biomass flux. A single unique biomass precursor reaction accounts for the multiple species’ biomass production in PGMM. In comparison, the GSMM-based community relies on precursor reactions within each species for their respective biomass production. Differences also surfaced in the prediction of certain biomass precursors, including higher fluxes for amino acids like cysteine and phenylalanine in PGMMs and lower fluxes for secondary metabolites such as cholate and phenol, alongside polysaccharide precursors (N-acetyl D-glucosamine and glucosamine). These variations could be attributed to a potential trade-off between accuracy and abstraction stemming from information loss.^48^

We further investigated the unique potential of PGMMs in exploring the metabolic landscape of the genus. We simulated PGMMs with distinct species probability vectors, replicating varying species proportions in a genus. The analysis of reaction and metabolite similarity between PGMMs illustrated the clustering of genera with similar functions as comparable to the phylogenetic tree, aligning with previous findings indicating shared functionalities among phylogenetically diverse organisms.^49^^,^^50^^,^^51^ Notable disparities between the reaction and metabolite similarity tree and metabolic flux bandwidth distance tree suggested that active metabolic fluxes may differ based on nutritional supplements and environmental factors compared to the putative functionalities observed within genera.^52^^,^^53^ Additionally, the clustering of *Prevotella* and *Bacteroides* in the flux bandwidth-based tree could be supported by the shared core protein similarity^54^^,^^55^ between the two genera despite their associations with different diets.^56^ Similarly, metabolic regulation clustering observed among opportunistic pathogens such as *Escherichia* and *Shigella* is consistent with their genetic similarity.^57^^,^^58^ Moreover, significant variations in the amino acid production potential of dysbiotic communities^20^^,^^59^ present evidence for the enhanced regulation of amino acid production in opportunistic pathogens. Ultimately, the flexible *Panera* PGMM proved to be a valuable resource for investigating the capabilities of microbial genera and customizing species composition within PGMMs, providing a significant advantage for studying core functionalities and niche development. Furthermore, these models can be employed to investigate context-specific genus similarity, providing insights into the functional relatedness of genera under specific conditions. Considering PGMM’s ability to comprehend the metabolic potential space of a genus in a given environment, it can serve as a scrutinizing step in formulating synthetic consortia for microbiome modulation strategies. Moreover, *Panera* can be instrumental in exploring the metabolic niche contribution of identified MAGs within their respective genera, by using good quality draft GSMMs built from MAGs, and understanding their metabolic variation under different environmental conditions.

Finally, we reported the principal utility of PGMM, which is to be a valuable tool in constructing microbial communities using incomplete taxonomic information. The evaluation of PGMM in a microbial community used two distinct datasets: (1) synthetic microbial abundance data with different community sizes and (2) metagenomics and metabolomics data collected from healthy individuals and CRC patients. Both analyses revealed that hybrid community models, which incorporate both GSMMs and PGMMs, offer predictions comparable to GSMM communities, surpassing the performance of PGMM or PanModel communities. As expected, PGMM communities outperformed PanModel communities in synthetic microbiome dataset. The predictability of hybrid models was particularly efficient with larger community sizes, demonstrating a better qualitative metabolic flux prediction in both 10-member and 50-member community models. The enhanced predictability observed can be attributed to the incorporation of species-level metabolic information alongside the genus-level data while characterizing a community. This approach might provide a more nuanced representation of metabolism within a community. Notably, previous studies that often-simplified 16S rRNA taxonomic information to the genus level for metabolic analysis can use hybrid model communities as a promising alternative without compromising data richness. This strategy is especially pertinent since 16S rRNA sequencing provides a combination of species and genus information. In addition, PGMMs can be tailored to incorporate prior probabilities if the information is available for a better accurate representation of a genus under a specific context. For example, suppose the adult gut microbiota is known to comprise 50% *Bacteroides fragilis*, 30% *Bacteroides vulgatus*, and 20% of the remaining species within *Bacteroides* genus. In that case, these prior probabilities can be applied to create a more precise model of the *Bacteroides* in the gut microbiota. Despite the variations in metabolic predictions, the adaptable PGMM and hybrid GSMM-PGMM communities demonstrate their significance in studying the metabolic abilities of microbial communities reconstructed from ambiguous amplicon sequencing data.

In summary, we have developed a unique framework, “*Panera*,” which can significantly reduce uncertainties in metabolic profiling of personalized microbial communities using ambiguous relative abundance data obtained from 16S rRNA sequencing analysis. The unique, flexible nature of the PGMM facilitates the examination of metabolic profiles at varying species compositions within a genus. This adaptability empowers the exploration of the metabolic landscape of genera and becomes instrumental in investigating the shared functionalities between genera and modulatory potential of genera within microbial communities. Furthermore, our study demonstrates that the hybrid community model, combining PGMMs and GSMMs, is a viable and efficient approach for capturing the capabilities of a microbial community without any information loss, even when encountered with uncertain taxonomic information.

### Limitations of the study

A limitation in constructing PGMMs from GSMMs lies in the quality of the PGMM, which is contingent upon the quality of source models. Additionally, these GSMMs should have a consistent standard annotation to ensure that the combined reactions function seamlessly as a proper metabolic model. This limitation constrains using the “*Panera*” algorithm to models obtained from a single source with uniform annotation. Nevertheless, the current availability of 7,302 curated strain-specific metabolic reconstructions, comprising 504 genera in AGORA2,^34^ presents a substantial resource for PGMM reconstruction. Since there is widespread availability of publicly accessible 16S rRNA sequencing datasets, the “*Panera*” algorithm is designed to focus on the microbiome characterized using amplicon sequencing. This helps to use the prior studies to dive deeper to unravel the potential links between metabolic dependencies and disease conditions. In addition, the existing database is designed to cater only for the models from the Virtual Metabolic Human (VMH) database, and the reaction and metabolite database has to be generated before employing GSMMs from different sources.

## STAR★Methods

### Key resources table

**Table:** 

REAGENT or RESOURCE	SOURCE	IDENTIFIER
**Deposited data**		

Metagenomics and Metabolomics of healthy and CRC patients	Yachida et al.^60^	Table with metagenomic data; Raw sequencing data: GenBank: DRA006684; GenBank: DRA008156
Repository of GSMMs – AGORA 1.03	Magnúsdóttir et al.^35^	https://www.vmh.life/files/reconstructions/AGORA/1.03/AGORA-1.03-With-Mucins.zip

**Software and Algorithms**		

CobraToolbox v3.0	Heirendt et al.^61^	https://github.com/opencobra/cobratoolbox
MATLAB R2022b	The MathWorks Inc.	https://www.mathworks.com
R version 4.0.1	R Foundation for Statistical Computing	https://www.R-project.org/
Microbiome Modeling Toolbox V2.0	Baldini et al.^36^	https://github.com/opencobra/cobratoolbox/tree/master/src/analysis/multiSpecies/microbiomeModelingToolbox/
Distributed FBA – Julia v1.6.8	Heirendt et al.^62^	https://github.com/opencobra/COBRA.jl
*Panera*	This study	https://github.com/RamanLab/Panera/

### Resource availability

#### Lead contact

Further information and requests for resources and reagents should be directed to and will be fulfilled by the lead contact, Karthik Raman (kraman@iitm.ac.in).

#### Materials availability

This study did not generate new unique reagents.

#### Data and code availability

• This paper analyzes existing, publicly available data. The links for the datasets are listed in the key resources table.
• All original code has been deposited at GitHub and is publicly available as of the date of publication: https://github.com/RamanLab/Panera/
• Any additional information required to reanalyze the data reported in this paper is available from the lead contact upon request.

### Experimental model and study participant detail

The presented analysis uses publicly available processed fecal metagenomic data collected from healthy individuals and CRC patients. The study does not generate new information for the existing data or involve the collection of new human samples. Instead, the investigation focuses on the re-analysis and interpretation of existing publicly accessible datasets.

### Method details

To mitigate the ambiguity in taxonomic resolution stemming from 16S sequencing in metabolic modeling and bridge the gap in creating a species-aware PGMM (PGMM), the ‘*Panera’* method is proposed. Figure 1 visually summarizes the PGMM reconstruction process. The figure also highlights the key distinctions between PGMMs and alternative models, along with their respective applications in microbial community modeling. The algorithm generates a PGMM from the existing strain/species-specific GSMMs in the AGORA database. AGORA (Assembly of Gut Organisms through Reconstruction and Analysis) is a database of semi-automatically curated genome-scale metabolic reconstructions of human gut microbes. AGORA-1.03 includes 818 metabolic reconstructions representing 1470 KEGG orthology identifiers (KO IDs), 227 genera and 14 different phyla. ModelSEED and KBase-based draft reconstructions of microorganisms from the annotated reference genome are gap-filled to ensure the reaction’s directionalities, mass, and balance charge. The gap-filled draft reconstructions are further refined with publications and comparative genomic analyses.

#### Formulation

Reconstruction of PGMM from species-specific GSMMs of a selected genus can be performed using the *‘Panera’* algorithm. The reconstruction pipeline employs three steps to produce a flexible PGMM: (i) Building a unified model from the reactions in all the species of a genus, (ii) Formulating biomass to represent all the species in a genus model, and (iii) Adding fields to accommodate the variation in species composition. The steps included in the PGMM reconstruction are illustrated in Figure S4 and detailed in this section.

##### Building a unified model from all the species genome-scale metabolic model of a genus

(1) A database of all metabolites and reactions in VMH models^63^ is retrieved from the Demeter pipeline.^64^ A separate database for the biomass reactions and metabolites of the species models is generated for the reconstruction (Table S1: Information of the species biomass reactions used in the model reconstruction).
(2) Reactions from the selected species GSMM models of a specific genus are extracted, and unique reactions (set of all the reactions) are identified to build a model.
(3) Unique reactions, except species biomass reactions, are integrated into a model using rBioNet. The fields such as rxnNames (reaction names), grRules (gene reaction association), compNames (Compartment where the reaction takes place - cytosol or Extracellular) and subsystems are added using a reaction and metabolite database.

##### Formulating biomass to represent the species in a genus model

(4) The biomass reaction for the pan-genera model is formulated as the linear combination of biomass reactions of individual species in the genus:

$$v_{panBiomass\;}=\sum_{i=1}^{n}v_{bio}^{i}*s_{i}$$

where $v_{panBiomass\;}$ is the biomass flux of the pan-genera model (Objective function), *n* is the number of species in the genus, $v_{bio}^{i}$ is the biomass flux of the $i^{th}$ species and $s_{i}$ is the coefficient for $i^{th}$ species, which implies the relative abundance or proportion of the microbial species in a community. The $s_{i}$ values can be adjusted to study the influence of a particular species in a genus. The reactions and metabolites associated with the ‘panBiomass’ and species biomass reactions are incorporated using biomass reaction and metabolite database. The default values of coefficients of species biomass ($s_{i}$) will be set to $\frac{1}{n}$. The default setting establishes an equal contribution from each species, and the coefficients can be adjusted to explore the distinct impact of a species.

(5) Duplicate reactions or metabolites and reactions/metabolites involved in futile cycles are removed from the PGMM if the removal does not impact the growth of the model.
(6) The refined pan-genus model is examined for growth by optimizing the model with biomass as an objective while constraining to a provided media condition.

##### Adding fields to accommodate the species composition variation

7. After PGMM refinement, a “reaction-species matrix”, a binary matrix representing whether the reaction is present (1) or absent (0) for an individual species, is combined as a field (‘rxnPresenceMat’) with the model.

(8) An ‘spList‘ field is incorporated into the model. Both ‘rxn-species matrix’ and ‘spList’ along with normalized ‘species probability vector’ will help filter the reactions to include in PGMM.

PGMM can be customized for a user-defined species composition using two key variables: (i) ‘species probability vector’, a vector of length n, a user-defined vector to reflect the estimated abundances of species in a community; and (ii) ‘rxn-species matrix’, a predefined matrix that encodes the reaction presence within a species. The product of these two variables determines whether the reaction is active in the model. A non-zero product indicates that the corresponding reaction is present in at least one species with a non-zero abundance, allowing it to be active within the model. Furthermore, the species probability vector plays a crucial role in incorporating compositional constraints into the biomass formulation. This formulation, in turn, influences the flux of internal and exchange reactions within the model.

#### Analysis of the reconstructed pan-genera metabolic model in analyzing species metabolic abilities

The reconstructed PGMM, which represents the universe of reactions and metabolites present in all the species within a genus, was used to perform *in silico* simulations using CobraToolbox.^61^ The PGMM was assessed through the prediction accuracy of the growth of individual species and the collective growth of all species within PGMM. The workflow is illustrated in Figure S5.

##### Simulation of metabolic capabilities of an individual species

We conducted initial simulations to explore the effect of including reactions from other species in PGMM while studying the metabolism of an individual species. Figure S5 illustrates the workflow employed for PGMM validation. Individual species models were derived from the PGMM using a species probability vector. To adapt the PGMM to specific species compositions, we adjusted the model based on the species probability/abundance. For instance, in the case of a PGMM representing a genus with five species, we simulated with a species probability vector indicating the presence of a single species at a time (Simulation 1: [1,0,0,0,0]; Simulation 2: [0,1,0,0,0] and so on).

A total of 150 species were selected for comparison of the metabolic abilities of customized PGMMs with GSMMs. Reactions with a reaction presence probability (product of species probability and reaction-species matrix) of more than zero were retained, while zero probability reactions were constrained to zero on their lower and upper bounds. The panBiomass reaction coefficients were adjusted to represent a species model, and the model was then subjected to FVA.^65^ The maximum flux of FVA was used as an indicator for the metabolic production capability of the model. Additionally, we analyzed the growth and maximum metabolite production potential of the species-specific GSMM. To evaluate the ability of PGMM to preserve the functionalities of a single species, we compared the metabolite production abilities between species-specific GSMM and modified PGMM under a given media condition. Jaccard distance between the maximum FVA values from GSMM and PGMM was evaluated to represent the qualitative variation by capturing the differences in the production of metabolites in the model, i.e., distinction in the metabolites with non-zero flux values. Meanwhile, the Euclidean distance between the maximum FVA of PGMM and GSMM was calculated to explain the quantitative variation, i.e., the magnitude of variation in the production flux value of the metabolites in the model. This distance metric provides insight into how much number of produced metabolites differs between the models. To ensure comparability across different models, we normalized the Euclidean distance by dividing it by the maximum value observed among all the models. In addition, to study the fraction of reactions exhibiting varying fluxes, we defined the stringent threshold of 10% flux variation between the comparable models.

##### Working of pan-genus metabolic model

The top-down approach of reconstructing PGMM aims to capture the genus-wide functionalities using species-level metabolic information. We assigned equal species probability as coefficients for biomass reactions in PGMM. For example, if a genus contains ten species, the coefficients for all species biomass reactants in the panBiomass reaction were set to 0.1, reflecting an equal contribution from each species. We generated a reference genus model with an equal abundance of species within a genus using GSMM and MMT v1. For comparative analysis, we then generated customized PGMMs and GSMM-based communities for 20 different genera retrieved from the AGORA database. FVA was performed on both the PGMMs and the genus models derived from GSMMs. By comparing the presence and magnitude of metabolite production across these models, we assessed the ability of PGMMs to represent the conserved and unique metabolic traits of a genus.

#### Application of pan-genus metabolic models in interpreting the metabolic landscape of a genus

Scouring the metabolic functional terrain of a genus could illustrate and cast light on its metabolic diversity trajectories and niche development.^53^ We analyzed the PGMM with varying species composition of a genus to explore their metabolic landscape (Figure S6A). The varying species combination representing the changing genera configurations was implied on the model by applying a species probability vector, which was generated by normalizing the sum of randomly generated values for each species within the genus to 1. The model was tailored to the given species composition by constraining the reaction bounds and species biomass coefficients in panBiomass.

Tailored PGMMs were subjected to FVA under different dietary conditions - European (EU) diet and Mediterranean diet (The constraints for the diet conditions were retrieved from VMH) and maximum flux was utilized from FVA to evaluate metrics to define the flux bandwidth of the metabolites. Two different metrics, (i) average maximum flux, which represents the mean of maximum flux of the metabolite production/consumption across different species composition and (ii) flux range, which explains the difference between the highest maximum flux to the lowest maximum flux observed for a metabolite across varying compositions were used to categorize the reactions into.

(1) No production - if both the averaged maximum flux and flux range are zero;
(2) Low varying reactions - if the averaged flux is non-zero and the flux range falls between 5% and 25%;
(3) Highly varying - if the flux range is greater than 50% and.
(4) Tightly regulated - if the flux range is within 5%.

#### Utility of pan-genus metabolic models in microbial community metabolic modeling

Metabolic modeling of metagenomics data-derived microbial communities presents a valuable tool for probing the hidden complexities of microbial associations and their metabolic interactions.^66^ Investigating the metabolic exchanges in a community unveils the interplay within the microbial species in a community and between the microbes and environment/host. In the current study, we substantiated the utility of PGMM in microbial community modeling by examining the metabolism of communities using synthetic and publicly available metagenome datasets. Given that PGMMs are species-aware, the unique functionality of those in creating communities with hybrid models are also analyzed. The workflow to infer how PGMMs could improve the insights about community interactions over GSMMs in synthetic and real metagenomic datasets is illustrated in Figure S6B.

##### Application of pan-genus metabolic model in analyzing metabolic capabilities of synthetic microbiota

###### Synthetic abundance data generation

To evaluate the applicability and efficiency of PGMMs in characterizing microbiota, we generated synthetic abundance data for various community sizes. We generated synthetic abundance data for 34 samples of a 10-member community and 15 samples of a 50-member community by randomly selecting ‘*k*’ strains from the pool of 818 AGORA metabolic reconstructions and assigning a random value to each strain. We performed data normalization, ensuring that the total abundances summed to 1. The normalized data were then grouped at the genus level to construct a genus-level abundance matrix. Additionally, we explored *hybrid* models that use taxa information resolved at both species and genus levels. Specifically, we conducted simulations using abundance data where 50% of the taxa were resolved to the species level, while the remaining were resolved only to the genus level (represented in Figure S6B).

###### Microbiota models from synthetic abundance data

The generated abundance data were utilized to construct the personalized microbiota models. MMT creates a template community model comprising all the species and/or genera in the dataset. Personalized models were then generated by adjusting species or genus biomass coefficients in the community biomass equation. We built four different community model types using (i) GSMM, (ii) PGMM derived from the present work, (iii) PanModel created using createPanModel of the MMT (PanModel), and (iv) hybrid models, where both GSMM and PGMM were incorporated. These models were compared against the widely used GSMM-derived communities.

All the community models were constrained to the European Diet, as reported in VMH.^63^ We performed FVA on the secretion and uptake fluxes of exchange metabolites within these diet-constrained models. We conducted the computational analysis with a high-level, multi-process and high-performance method, ‘distributed FBA’ in Julia v1.6.8^62^^,^^67^ combined with CPLEX solver v12.8 to accommodate the larger number of microbial members in a community. Jaccard and Euclidean distances were used to assess differences in metabolites with non-zero flux and the variation in flux value magnitude between the community models, respectively. This evaluation aimed to elucidate the ability of PGMMs to capture species-model metabolic inference within the microbial community and to determine the advantages of our metagenomics-informed PGMM over the lumped PanModel.

###### Comparison of metabolic prediction of genome-scale metabolic model and pan-genus metabolic model with metabolomics data

We investigated the potential of PGMMs in characterizing personalized gut microbiome metabolic communities from colorectal cancer (CRC) patients. We leveraged study^60^ that provided both metabolomic and metagenomic data from gut microbiome samples of healthy individuals and CRC patients. The personalized communities were built using different model sources (GSMM, PGMM, PanModels and hybrid (GSMM and PGMM)) and the FVA of those communities were carried out for the comparison. Due to computational demands associated with constructing personalized microbial communities for all samples using four different model sources (∼4N), only a subset of samples was used for the analysis. Of the 406 subjects, we selected two different sets of 5% of samples (around 20) encompassing both healthy and CRC samples for a comparative analysis of microbial community functionality using various community modeling approaches in conjunction with metabolomics data (Detailed metadata is provided in Table S7D). We preprocessed the normalized abundance values of microbial species in the selected samples by removing rare taxa, defined as taxa with an abundance lower than 10^−3^. Additionally, we mimicked amplicon sequencing data by converting approximately 50% of the species information to the genus level, enriching the abundance table with both species and genus-level information.

We constructed personalized community models for each sample with different source models (GSMM, PGMM from our algorithm, PanModel from MMT and hybrid approach) based on the processed abundance table. Subsequently, we comprehensively analyzed the FVA of exchange reactions in these community models using 'distributed FBA' in Julia to assess their metabolite production potential. To gauge the accuracy of our predictions, we compared the net flux of exchange metabolites (Sum of secreted and uptake fluxes) from the simulated microbial communities with the actual metabolomic data. While the reference study reported the concentration of 450 metabolites to characterize the metabolomics of a community, the comparison focused on a more targeted set of approximately 290 metabolites. These metabolites represent the unique set of metabolites produced across all analyzed communities and overlap with the metabolites in the metabolomics study. We employed Jaccard and Euclidean distance metrics to evaluate the accuracy of our predictions and identify potential errors in predicting metabolic capabilities. All the model communities were compared against the standard GSMM-based microbial community model predictions for estimating quantitative variation.

### Quantification and statistical analysis

All the community model simulations were carried out using MMT in CobraToolbox v3.0 and MATLAB R2022b. The paired t-test is employed to calculate the significance of metabolic prediction variation between the communities from different model sources (PGMM, PanModel and hybrid) with respect to GSMM-based communities using ‘*ggpubr’* package. The level of significance is represented as follows: ns - not significant; ∗ - p ≤ 0.05; ∗∗ - p ≤ 0.01; ∗∗∗ - p ≤ 0.001 and ∗∗∗∗ - p ≤ 0.0001. The figures were generated using BioRender, InkScape and the *ggplot2* package in R (version 4.0.1). All the data are represented as single data points in the figures.

## Supplemental information

Document S1. Figures S1–S6

Table S1. Information on the species biomass reactions used in the model reconstruction, related to Figure 1B

Table S2. Metabolic information of the built PGMMs, related to Figure 1C

Table S3. Metric to compare the PGMM extracted species model and individual GSMM, related to Figure 2A

Table S4. Metric to compare the equal probability imposed genus model and GSMM-based genus model, related to Figure 2B

Table S5. Metagenomic composition and prediction comparison metrics of 10-member synthetic communities, related to Figures 4A and 4B (A) Metagenomic composition of the communities.(B) Qualitative (Jaccard distance) comparison between different model-based communities with respect to GSMM. (C) Quantitative (Euclidean distance) comparison between different model-based communities with respect to GSMM.

Table S6. Metagenomic composition and prediction comparison metrics of 50-member synthetic communities, related to Figures 4A and 4B (A) Metagenomic composition of the communities. (B) Qualitative (Jaccard distance) comparison between different model-based communities with respect to GSMM. (C) Quantitative (Euclidean distance) comparison between different model-based communities with respect to GSMM.

Table S7. Metagenomic composition and prediction comparison metrics of real healthy and colorectal cancer (CRC) communities (two sets of analysis), related to Figures 4C and 4D (A) Metagenomic composition of the communities. (B) Qualitative (Jaccard distance) comparison between different model-based communities with respect to Metabolomics data. (C) Quantitative (Euclidean distance) comparison between different model-based communities with respect to GSMM. (D) Metadata of all the healthy and CRC samples. (E) Qualitative (Jaccard distance) comparison between different model-based communities with respect to Metabolomics data (Second Set). (F) Quantitative (Euclidean distance) comparison between different model-based communities with respect to GSMM (Second Set).
