# Supplementary material for: Panera: An innovative framework for surmounting uncertainty in microbial community modeling using pan-genera metabolic models
Source: iScience. 2024 Jun 22;27(7):110358. doi: 10.1016/j.isci.2024.110358 (PMC11292516; doi:10.1016/j.isci.2024.110358)
Supplement: Document S1. Figures S1–S6 [file mmc1.pdf]

iScience, Volume *27*

Supplemental information

Panera: An innovative framework for surmounting uncertainty in microbial community modeling using pan-genera metabolic models

Indumathi Palanikumar, Himanshu Sinha, and Karthik Raman

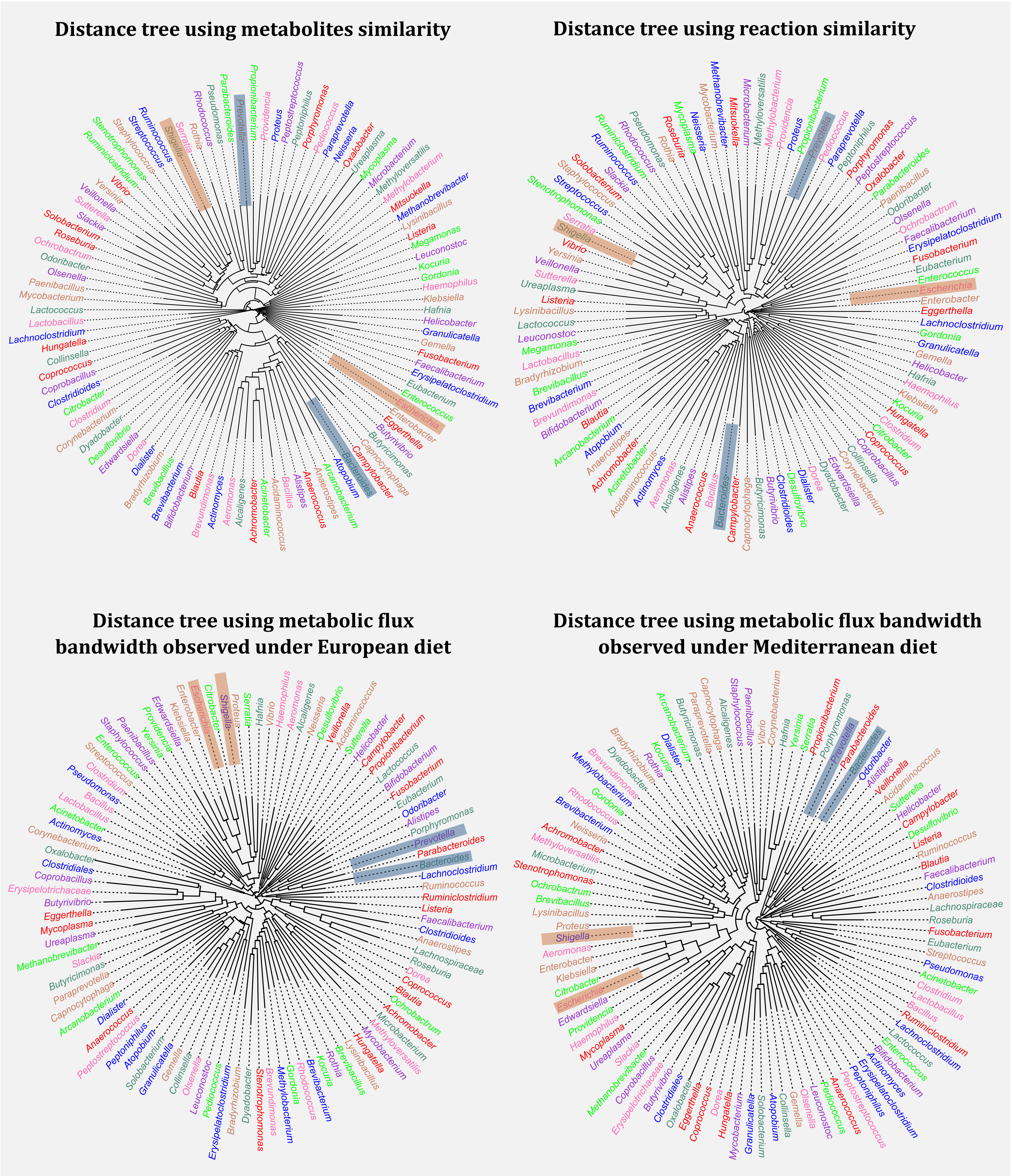

Supplementary Figure S1: Analysis of the metabolic and reaction similarity based on the GSMM under specific diet conditions, Related to Figure 3

A & B) Distance tree based on the Jaccard similarity between the reaction/ metabolite presence between the PGMMs. The color of the text within the tree indicates the phylum of each genus.

C & D) Distance tree genrated by comparing the metabolic flux bandwidth type observed for genus under (C) European diet and (D) Mediterranean diet

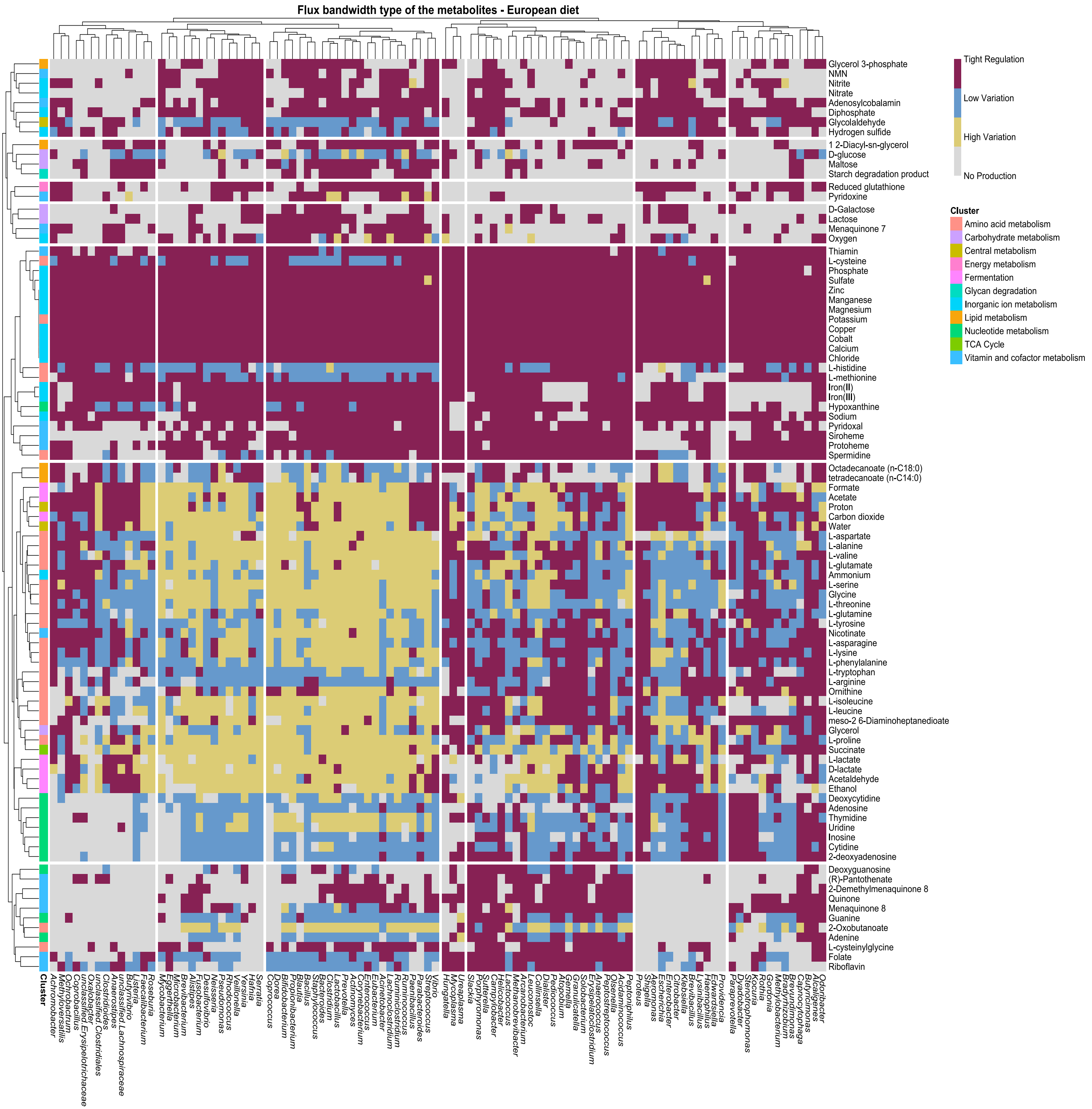

Supplementary Figure S2a: Flux bandwidth patterns of exchange metabolite production for various genera under European diet, Related to Figure 3

Rows represent individual exchange metabolites, while columns represent individual genera. The figure excludes the metabolites which are not produced in more than 90% of the genera. Hierarchical clustering is employed to group both genes and metabolites based on their similarity in bandwidth patterns. The color within the heatmap reflects the metabolic flux bandwidth for a particular metabolite-genus combination. Subsystem information associated with the reactions responsible for metabolite production is represented on the right side of the plot.

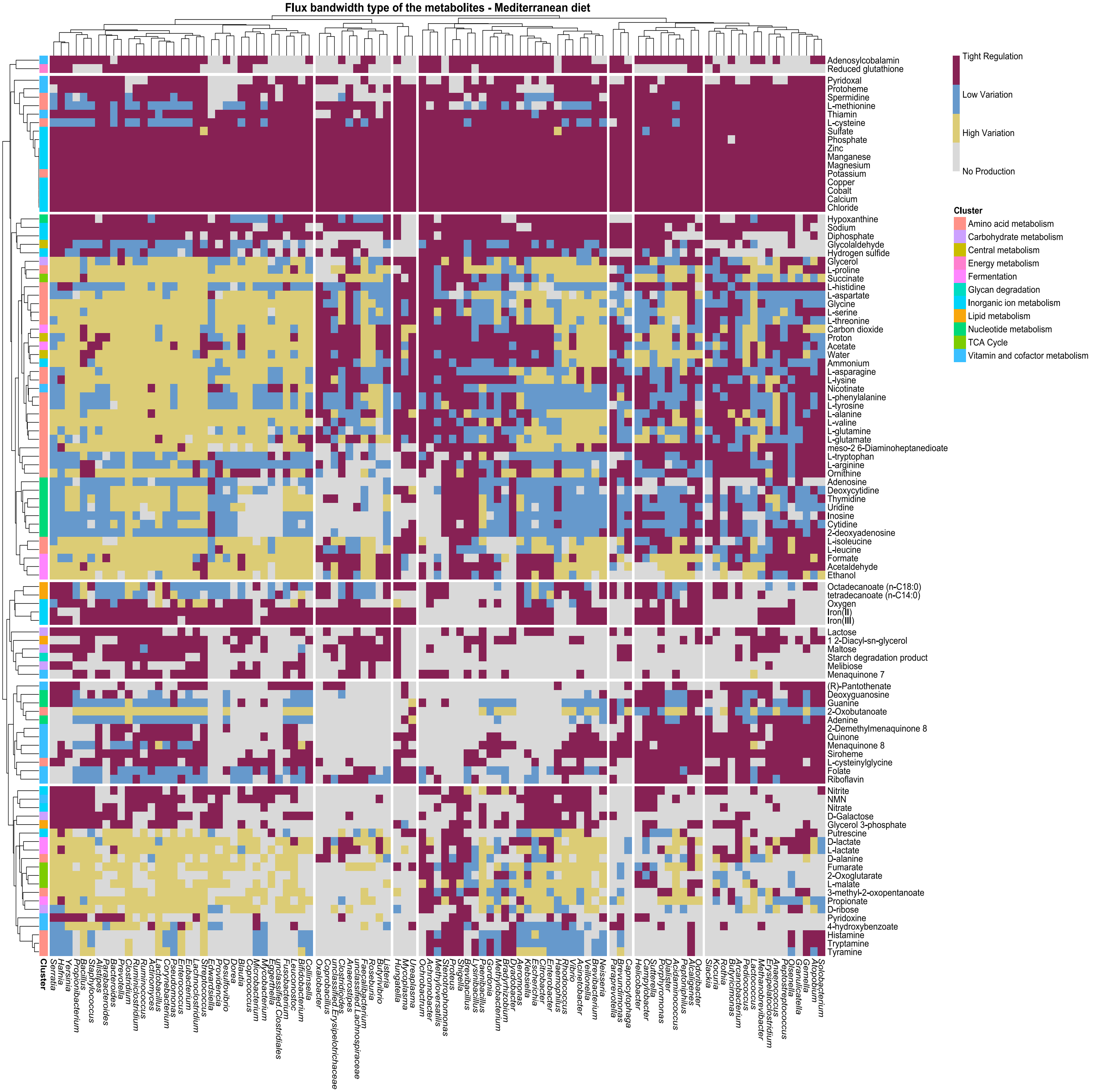

Supplementary Figure S2b: Flux bandwidth patterns of exchange metabolite production across genera for a Mediterranean diet, Related to Figure 3

In this representation, rows correspond to exchange metabolites, and columns depict the individual genera. The figure excludes the metabolites which are not produced in more than 90% of the genera. Hierarchical clustering is employed to group both genes and metabolites based on their bandwidth patterns. The color within the heatmap reflects the metabolic flux bandwidth for a particular metabolite-genus combination. Subsystem information associated with the reactions responsible for metabolite production is represented on the right side of the plot.

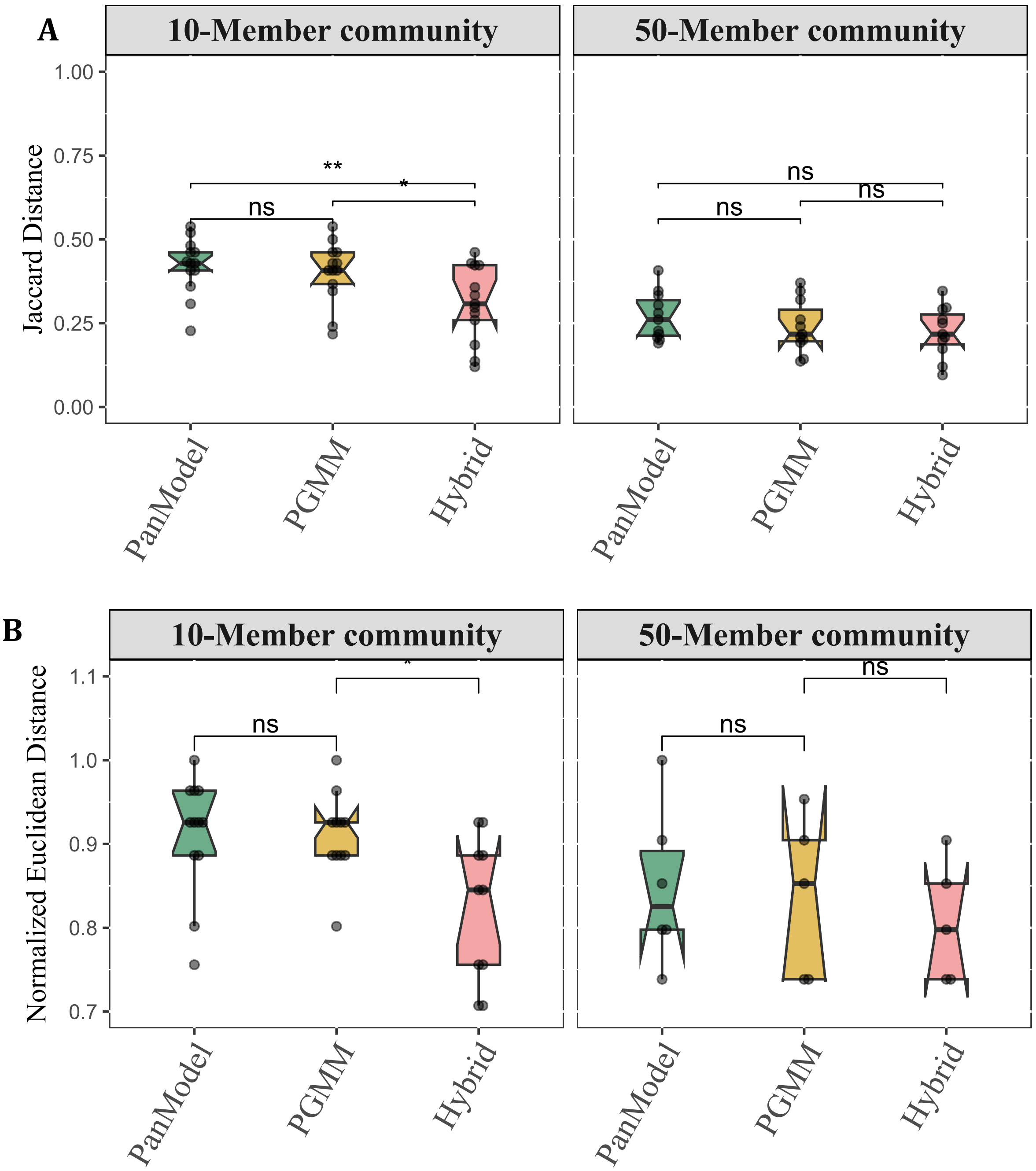

Supplementary Figure S3: Comparative qualitative and quantitative variation analysis for demonstrating PGMM applicability in community modelling, Related to Figure 4

A comparative analysis of qualitative (Jaccard distance) and quantitative (Normalized Euclidean) differences between the metabolic uptake potential for synthetic dataset (A and B). The analysis is employed for different community types, including PGMM (Panera-derived Pan-Genus Metabolic Model), PanModel (Pan-Genus Metabolic Model constructed with 'CreatePanModels' in the CobraToolBox suite), and Hybrid communities (combining GSMM and PGMM). The values are calculated in relation to GSMM-based community models.

(A) Highlights qualitative differences in metabolic diversity and (B) quantitative differences (variation in metabolite uptake flux) when compared to GSMM-based communities for both 10 and 50-member synthetic communities.

The reported significance is calculated based on the paired t-test and the level of significance is represented as follows: ns - not significant; * - p <= 0.05; ** - p <= 0.01; *** - p <= 0.001 and **** - p <= 0.0001.

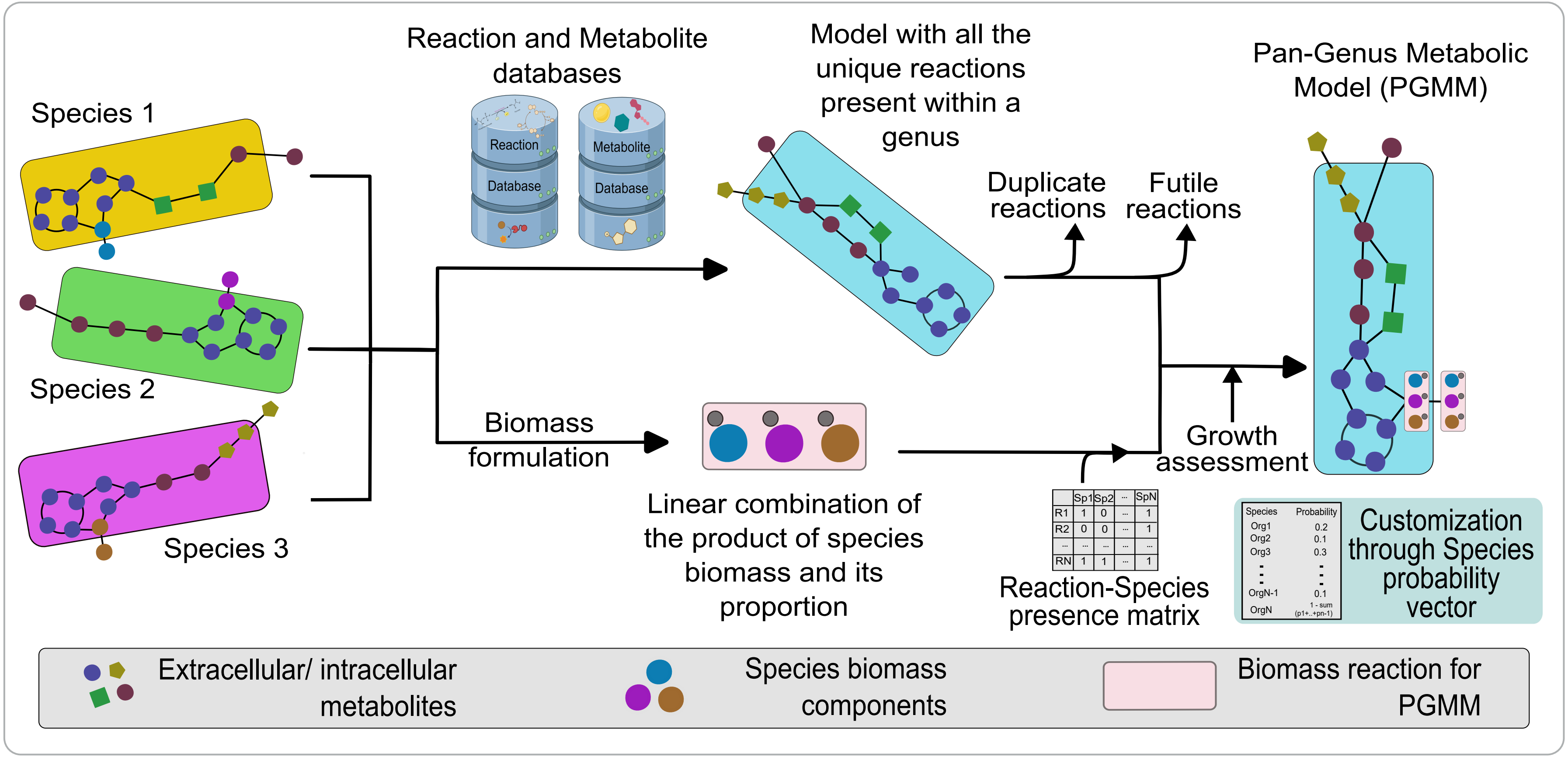

Supplementary Figure S4: Overview of *'Panera'* methodology , Related to Figure 1

Detailed methodology workflow of the Pan-Genus Metabolic Model (PGMM) reconstruction through ‘*Panera*’ methodology

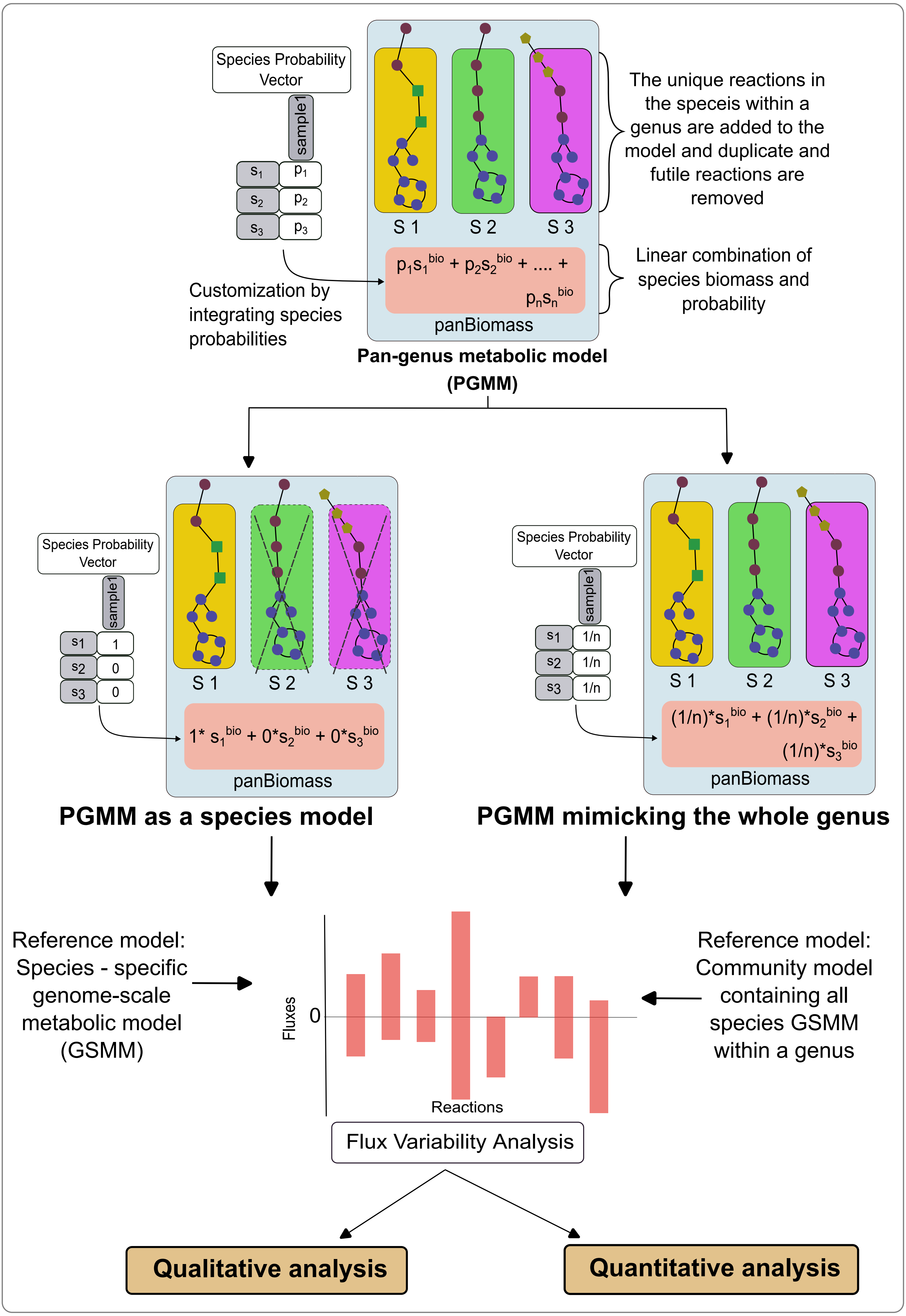

Supplementary Figure S5: Illustration of PGMM validation by replicating the individual species and whole genus, Related to Figure 2

PGMM is customised to extract the individual species and simulate an equal abundance-imposed genus model using the species probability vector. The qualitative and quantitative analysis using the maximum flux of the exchange metabolites from Flux Variability Analysis is explained in detail in the Methods section (5.2.1)

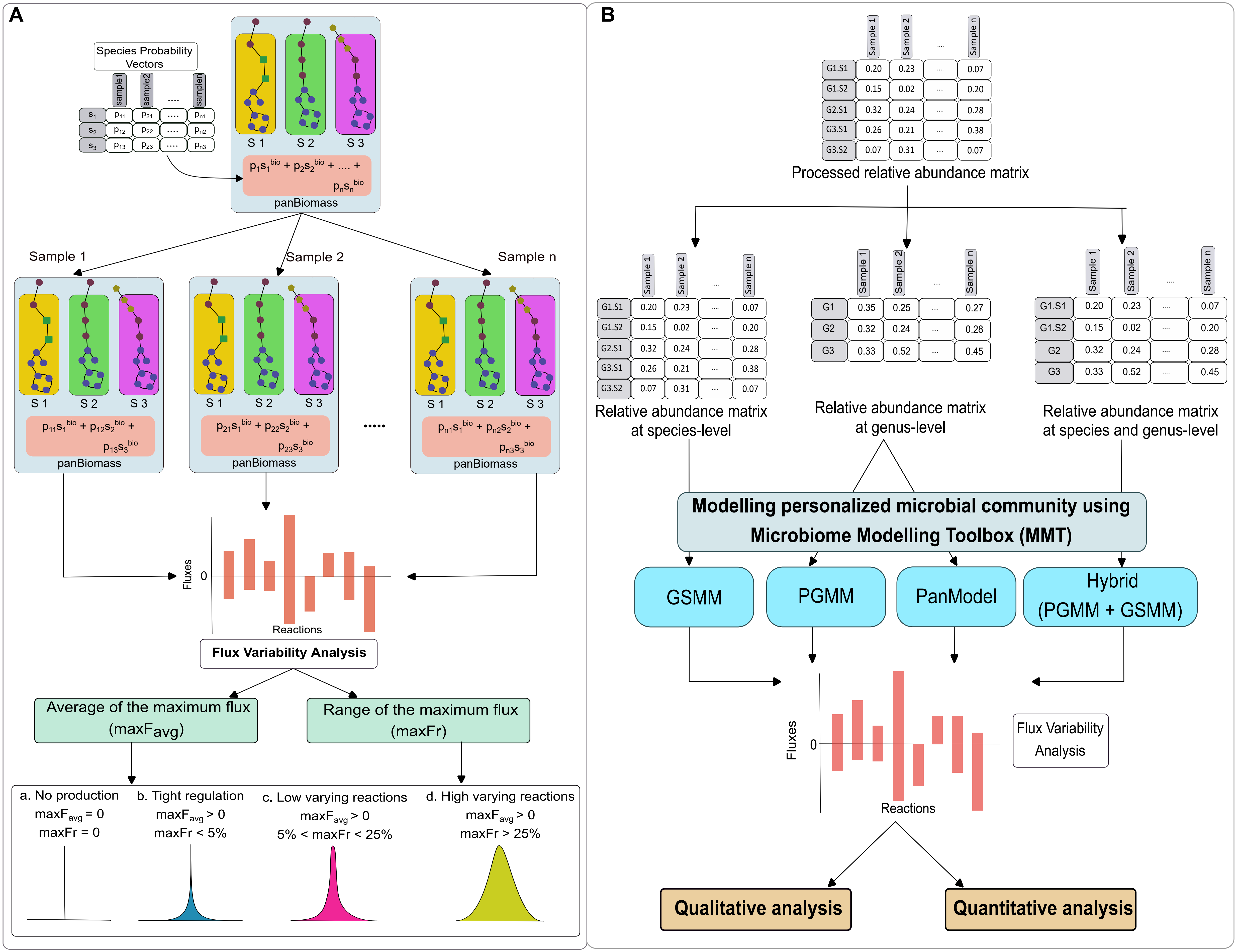

Supplementary Figure S6: Workflow for demonstrating the applicability of PGMM, Related to Figure 3 & 4

(A) in exploring the metabolic capabilities of a genus by simulating different species composition within a genus

(B) in modelling microbial communities. The microbial community modelling is carried out for both synthetic abundance data of different community sizes (10 and 50) and CRC-based metagenomics data. The abundance table is modified by aggregating at the genus level for modelling PGMM and PanModel-based communities and aggregating only 50% of the data to the genus level for modelling hybrid communities.
